# NMR Structure and Dynamics of the Resuscitation Promoting Factor RpfC Catalytic Domain

**DOI:** 10.1371/journal.pone.0142807

**Published:** 2015-11-17

**Authors:** Vincenzo Maione, Alessia Ruggiero, Luigi Russo, Alfonso De Simone, Paolo Vincenzo Pedone, Gaetano Malgieri, Rita Berisio, Carla Isernia

**Affiliations:** 1 Dipartimento di Scienze e Tecnologie Ambientali Biologiche e Farmaceutiche, Second University of Naples, via Vivaldi 43, 81100, Caserta, Italy; 2 Istituto di Biostrutture e Bioimmagini, CNR, Via Mezzocannone 16, 80134, Napoli, Italy; 3 Centro Interuniversitario di Ricerca sui Peptidi Bioattivi—University of Naples “Federico II”, Via Mezzocannone 16, 80134, Napoli, Italy; 4 Faculty of Natural Sciences, Department of Life Sciences, Imperial College, 603 Sir Ernst Chain Building-South Kensington Campus, London, United Kingdom; Russian Academy of Sciences, Institute for Biological Instrumentation, RUSSIAN FEDERATION

## Abstract

*Mycobacterium tuberculosis* latent infection is maintained for years with no clinical symptoms and no adverse effects for the host. The mechanism through which dormant *M*. *tuberculosis* resuscitates and enters the cell cycle leading to tuberculosis is attracting much interest. The RPF family of proteins has been found to be responsible for bacteria resuscitation and normal proliferation. This family of proteins in *M*. *tuberculosis* is composed by five homologues (named RpfA-E) and understanding their conformational, structural and functional peculiarities is crucial to the design of therapeutic strategies.Therefore, we report the structural and dynamics characterization of the catalytic domain of RpfC from *M*. *tubercolosis* by combining Nuclear Magnetic Resonance, Circular Dichroism and Molecular Dynamics data. We also show how the formation of a disulfide bridge, highly conserved among the homologues, is likely to modulate the shape of the RpfC hydrophobic catalytic cleft. This might result in a protein function regulation via a “conformational editing” through a disulfide bond formation.

## Introduction

Tuberculosis is an infectious bacterial disease caused by various strains of *mycobacteria*, most commonly *Mycobacterium tuberculosis*.

This latter is a very successful pathogen that can persist in the human host regardless of a strong immune response; a large part of the human population, is in fact, latently infected with *M*. *tuberculosis*. The bacillus resides in the host in a dormant form creating an enduring pool of future disease and contagion.

Although the interactions between *M*. *tuberculosis* and the host organism resulting in chronic or latent infection remain elusive, the mechanism through which this bacterium "awakes" from its dormant state is currently attracting much of the research activity in this area.

The factors found to be responsible for the resuscitation of the dormant bacteria, allowing them to proliferate normally, belong to the so called Rpf (Resuscitation promoting factors)protein family. This family of proteins in *M*. *tuberculosis* is composed by five homologues named RpfA-E [1]. Rpfs contain a transglycosylase-like domain and have been proposed to act as peptidoglycan hydrolases that alter the mechanical properties of the cell wall and favour cell division and/or release of anti-dormancy factors [2].

All Rpfs share a 70 amino acid catalytic domain and present N-terminal signal sequences that indicate that the proteins are translocated to an extracellular location. Individual *rpf A-E* genes were at first considered to be unnecessary for *M*. *Tuberculosis* growth both *in vitro* and *in vivo*[3, 4]. This initially suggested some functional redundancy between the genes that, later was demonstrated to be not straightforward, as mutants bacteria lacking three of five *rpf* genes were found to be defective for growth *in vivo* and for resuscitation *in vitro*[5]. This clearly indicated that the biological function of the five Rpfs is not completely redundant. For instance, recent *in vivo* studies have highlighted a different role for RpfB: its deletion produces delayed reactivation from latent tuberculosis [6]. In 2005, Cohen-Gonsaud and co-workers determined the NMR solution structure of the RpfB catalytic domain, showing that it has a lysozyme-like fold[7]. Later, the crystal structure of a major portion of RpfB was also determined [8] while a synergy between RpfB and RipA (resuscitation-promoting factor interacting protein) in peptidoglycan degradation and in the resuscitation of the dormant mycobacteria has been recently documented [9]. Also recent is the crystal structure determination of the RpfE catalytic domain[10] demonstrating that the protein adopts the characteristic Rpf fold, as observed in RpfB, but with some distinctive features, especially in terms of electrostatic potential surface of the catalytic cleft. This latter feature has suggested a different functional specificity of RpfE protein.

Also the X-ray structure of the RpfC catalytic domain has been determined[11], and also in the case of this structure the high degree of structural conservation within the Rpf family has emerged.For these reasons the exploration of the differences within these mycobacterial paralogues in terms of dynamics is certainly needed.

It is well established that the overall folds of proteins are generally very similar in solution and in the crystalline environment[12, 13]. However, details of the structure may differ, especially when it comes to the conformations of loops and surface side-chains. In fact, while coordinate accuracy of bio-molecular X-ray structures routinely reaches sub-angstrom levels, crystallographic experiments cannot directly prove the dynamics of bio-molecules and can capture their structural heterogeneity only indirectly [14, 15]. Internal protein dynamics can potentially affect protein function through a variety of mechanisms. In particular, in protein-protein and protein-ligand interaction processes the protein dynamics is fundamentally linked to function. For these reasons the description of the differences within this redundant protein family in terms of dynamics can provide important insights in the functional properties of resuscitation factors.

Therefore, we here report the structural, dynamic and functional characterization of the catalytic domain of RpfC from*M*. *tubercolosis* (RpfCc) using the combination of Nuclear Magnetic Resonance (NMR), Circular Dichroism (CD) and Molecular Dynamics (MD) simulations data. In particular, since the crystal packing may introduce conformational bias[16–18], we first analyzed the x-ray structure using the experimental NMR data (chemical shifts).Our analysis confirmed that the conformation observed in the crystal state belongs to the conformational ensemble in solution and also suggested that RpfCc is characterized by regions with conformational heterogeneity. The solution NMR structure of RpfCc shows the presence of a globular fold similar to that observed in the crystal state, albeit with small but significant differences in the three loop regions. Therefore, to appreciate the functional proprieties of RpfCc, understanding the connection between the three-dimensional structure and dynamics, we performed a backbone dynamics study and a conformational analysis of RpfCc using NMR and MD simulations techniques. Additionally, the analysis of the pH effect on the protein structure suggested that RpfCc may modulate its function by the redox-sensing switch, in response to changes of external environmental conditions. The structural and dynamic features outlined by our characterization are then compared with the other characterized members of this protein family.

## Materials and Methods

### Cloning, Expression and Purification

Oligonucleotide primers were synthesized by Primm (Milano, Italy) and were designed to amplify the nucleotide sequence corresponding to the the catalytic domain of RpfC (residues Gly68–Leu148 of UniProt RPFC_MYCTU) by Polymerase Chain Reaction (PCR) using genomic DNA of *Mtb* as a template (H37Rv strain). NcoI/HindIII-digested fragments were cloned into the expression vector pETM-11 to generate a recombinant protein containing a cleavable N-terminal poly-His tag for metal affinity purification. The resulting positive plasmid was used to transform *E*. *coli* Star BL21 (DE3) competent cells. Transformed cells were cultured overnight in Luria- Bertani broth with 50 mg ml^-1^ kanamycin at 37°C. For the production of isotope-labeled samples (^15^Nand ^15^N/^13^C), the culture was seeded in 1:100 volume ratio either in 1 l of minimal media (M9) containing 422 mM Na_2_HPO_4_, 220 mM KH_2_PO_4_, 85.5 mM NaCl, 186.7 mM of ^15^N ammonium chloride, 1 mM MgSO_4_, 0.2 mM CaCl_2_, 1 ml Thiamine 40 mg ml^-1^, and 0.3% final of ^13^C-glucose. Culture was grown at 37°C in a shacking incubator, induced with 0.8 mM Isopropyl b-D-1-thiogalactopyranoside (IPTG), and grown at 22°C for 16 h for protein production. *E*. *coli* cells were harvested by centrifugation at 6000 rpm for 20 min, and the pellet was resuspended in a buffer containing 300 mM NaCl, 50 mM Tris-HCl, 10 mM imidazole, 5% (v/v) glycerol and complete protease inhibitor cocktail (Roche Diagnostic), pH 8, and lysed by sonication on ice. The cell lysate was centrifuged at 16,500 rpm at 4°C for 30 min, and the supernatant was loaded on Ni^2+^- derivatized HisTrap columns (GE Healthcare). A linear gradient of imidazole was applied to elute the protein, which was then dialyzed against a buffer containing 150 mM NaCl, 50 mM Tris-HCl, 2% glycerol, pH 8.0 at 4°C for 16 hours and digested with Tobacco Etch Virus endopeptidase (TEV) protease to remove the 6X His tag.

This sample was further purified by a second Ni^2+^ affinity chromatography. The protein was concentrated using a centrifugal filter device (Millipore) with a cut-off of 3kDa. Protein concentrations were measured using absorption at 280 nm and resulted to be 1 mM for ^15^N RpfCc and 0.8 mM for ^15^N-^13^C RpfCc. The protein obtained has as an additional sequence of three residues (GAM) at N-terminal region due to the TEV cleavage. Therefore, we number the protein structure from residue Gly1, while the Gly4 corresponds to Gly68 in the UniProt entry.

### Circular Dichroism (CD) experiments

Protein samples were prepared in 4 mL of 20 mM phosphate buffer containing 0.15 M NaCl at pH 7.0. Samples in reducing conditions were prepared in the same buffer adjusted to pH 5.0 and 2.0 in presence of 2mM TCEP.CD spectra were recorded with a Jasco J-815 spectropolarimeter equipped with a Peltier temperature control system (Model PTC-423-S). Far-UV measurements were carried out on 25 μM of RpfCc protein using a quartz cuvette with a 1 cm path-length in the 200–260 nm wavelength range with a data pitch of 1 nm. All data were recorded with a bandwidth of 1 nm with a scanning speed of 50 nm min^-1^ and normalized against reference spectra to remove the background contribution of buffer. The thermal denaturation was carried out by measuring CD spectra at 5 K intervals from 278 K to 368 K. After the final measurement, the sample was cooled back to 278 K and a final spectrum collected. The spectra obtained were analyzed monitoring the molar ellipticity changes at 222 nm as a function of temperature increase. The data obtained were fitted into two-state folding model.

### NMR spectroscopy

All the NMR experiments were acquired at 298 K on a Varian Unity INOVA 500-MHz at Department of Environmental, Biological and Pharmaceutical Sciences and Technology of the Second University of Naples, using the standard pulse sequences. NMR samples consisted of 0.8–1 mM uniformly ^15^N or ^15^N-^13^C labeled protein dissolved in 20 mM sodium phosphate (pH 6.9), 0.15 M NaCl, 0.02% sodium azide and 10% ^2^H_2_O. The assignment of ^1^H and ^15^N resonances was achieved using a suite of heteronuclear 2D and 3D spectra: ^1^H-^15^N-HSQC, 3D [^1^H, ^15^NH]-TOCSY-HSQC, and 3D [^1^H, ^15^NH]-NOESY- HSQC. The assignment was extended to ^13^C resonances and globally assessed by analyzing (H)CCH-TOCSY and triple resonance (HNCO, HNCACB, CBCA(CO)NH) experiments. NOEs were evaluated from 3D ^15^N- and ^13^C-edited NOESY spectra. All NOESY spectra have been acquired with a mixing time of 90 ms. The relaxation parameters (longitudinal relaxation rate R1, transversal relaxation rate R2 and hetero-nuclear NOE) were evaluated by recording and analyzing the following set of experiments: Inversion Recovery ^1^H-^15^N HSQC with 7 different values of relaxation delay (0.01, 0.05, 0.1, 0.2, 0.3, 0.4, 0.6 s) for the evaluation of R1; Spin Echo ^1^H-^15^N HSQC with 7 different relaxation delay (0.01, 0.03, 0.05, 0.07, 0.01, 0.11, 0.13 s) for the evaluation of R2; two ^1^H-^15^N HSQCs for the evaluation of the ^1^H-^15^N NOE, in one the protons were unsaturated and in the other were saturated for 3s utilizing a 10kHz pulse.All NMR relaxation experiments were acquired twice to assess the reproducibility of the data.The hydrodynamic proprieties were estimated using the translational diffusion coefficient (Dt) measured by Pulsed-field gradient spin-echo DOSY experiments [19]. The R_h_ was estimated from the Stokes-Einstein equation: (K_B_T)/6πηD_t_, where K_B_ is the Boltzmann constant, T is the temperature in Kelvin and η is the viscosity of the solution in Pa s. The rotational correlation time (τc) was estimated, considering a spherical globular protein, through the hydrodynamic radius (Rh) from the Stokes-Einstein equation: τc ~ (4ηπR_h_^3^)/ 3 K_B_T. The hydrodynamic proprieties (D_t_, R_h_) were also evaluated from the 3D NMR conformers using the software HYDROPRO[20–22]. A correction factor was introduced to keep in count the major viscosity of the solution 90% and 10% ^2^H_2_O.

### Processing and analysis of the spectra

NMR experiments were processed using the software Varian (VNMR 6.1B). Proton chemical shifts, ^13^C and ^15^N were calibrated indirectly, using an external reference. Each recorded FID was multiplied by an appropriate weighting function, generally a “square shifted sine bell” and the “data points” were zero filled before the Fourier transform. The program CARA[23] was utilized to analyze and to assign the spectra. The data for the measurement of relaxation parameters were Fourier transformed after application of a cosine-squared apodization function to yield a matrix of 2048 (F2) * 512 (F1) data points.Peak intensities were manually measured rather than peak volume for the calculation of the relaxation times and NOE values. R1 and R2 rates were obtained by the fitting the peak intensities at multiple relaxation delays to the equation: *I* = *I*_0_ *e* ^(−*Rt*)^ [24] using GraphPad Prism software.

Uncertainties in R2 and R1 were obtained from the error fit. ^1^H-^15^N steady state NOEs were calculated as the ratio of ^1^H-^15^N correlation peak volume in the spectra acquired with and without the proton saturation during the five seconds recycle delay and their uncertainties were set to 5%. Model free parameters (S_2_, R_ex_ and τe) were estimated using the software Dynamics[25]. Residues with large-amplitude fast internal motions were excluded from the calculation. Among the remaining residues, those with significant conformational exchange on the microsecond to millisecond time scale were also excluded.

### Chemical shifts prediction

The chemical shift predictions for the X-ray structure were performed of RpfCc using the 4DSPOT software[26]. As the prediction tool requires the addition of explicit hydrogens before a calculation can be performed, we protonated each conformer in accordance with H++[27] pka predictions. The prediction of the chemical shifts from the X-ray structure was achieved as reported in the protocol presented in Lehtivarjo *et al*. [28]. The quality or Q factor was calculated using the equation presented in Cornilescu *et al*. and reported below:

$$\text{Q}=\text{rms}\;\left({\Delta \delta }^{\text{meas}}-{\Delta \delta }^{\text{pred}}\right)/\;\text{rms}\left({\Delta \delta }^{\text{meas}}\right)$$

where δ^meas^ is the measured chemical shift and δ^pred^ is the chemical shift predicted on the base of the X-ray structure.

### Structure calculation

The cross-peaks of the 3D ^1^H-^15^N NOESY-HSQC and the 3D ^1^H-^13^C-NOESY-HSQC recorded in H_2_O were manually integrated using the program XEASY[23]. The volumes were transformed in upper limit distance constraints by the CYANA routine CALIBA, using the functions V = A/r^6^ for the backbone and V = B/r^4^ for the side chains, where V is the peak volume and r is the corresponding distance. The distances thus obtained were used by GRIDSEARCH to generate a set of allowed dihedral angles; at the end, ANNEAL calculated the structure using the torsion angles dynamic. Protein structures were calculated using1030 inter-proton distance constraints, derived from NOESY experiments, and 120 dihedral angle restraints obtained by TALOS+ software [29](Table 1). A total of 100 structures was calculated, and the 20 conformers with the lowest CYANA target function were further refined by means of unrestrained energy minimizations with the program SPDB[30]. The small number of residual constraint violations (Table 1) indicates that the input data represent a self-consistent set and that the constraints are well satisfied in the calculated conformers. The global rmsd value calculated for the backbone atoms of the region 9–78 (Table 1) shows that an overall high precision of the structure determination has been achieved.

**Table 1:** NMR structural statistics.

NMR constraints	
Distance	1030
Intraresidue	539
Sequential (|*i*–*j*| = 1)	220
Medium-range (|*i*–*j*| < 5)	132
Long-range (|*i*–*j*| ≥5)	139
TALOS+ Dihedral angle restraints	120
Structure statistics	
Rms deviations from idealized covalent geometry	
Bond length (Å)	0.015
Bond angle (°)	1.9
Average rms deviation from current reliable structures (rms Z scores, null deviation = 1)	
Bond length (Å)	0.807 ± 0.007
Bond angle (°)	0.976± 0.015
Rms deviations from distance restraints(Å)	0.0046 ± 0.0005
Rms deviations from dihedral restraints (°)	0.104 ± 0.018
CYANA target function (Ǻ^2^)	0.57
AMBER Kcal/mol	
Total	-504 ± 19
Van der Waals	- 469 ±7
Electrostatic	- 319 ± 17
Coordinate precision	
RMSD from mean structure (residue 9–78) (Ǻ)	
All backbone atoms	0.634
All heavy atoms	1.034
Ramachandran analysis (residue 9–78), % residues	
Most favored regions	84
Additional allowed regions	14.9
Generously allowed regions	1.1
Disallowed regions	0

The structures were visualized and evaluated using the programs MOLMOL and PROCHECK-NMR [31].

### Molecular Dynamics (MD) Simulations

Simulations were performed using GROMACS [32] software. The Amber99SB [33] force field was used with explicit TIP3P water[34]. All simulations used a 2fs inner step and were carried out in NVT ensemble using a Nose-Hoover thermostat after equilibration to constant box volume in the NPT ensemble. The simulations were carried out with periodic boundary conditions at a constant temperature of 298 K. A rectangular box was used to accommodate the protein, water molecules, and ions. The system considered for RpfCc included 4261 water molecules.

## Results and Discussion

### Chemical shift assignment and analysis of the X-ray structure using NMR data

In order to complement the available structural data with an accurate description of the conformational space sampled by the protein in solution, we performed a structural and dynamics characterization of the RpfCc protein via Nuclear Magnetic Resonance and Molecular Dynamics techniques.

Resonance assignments for backbone H_N_, ^15^N, Cα and CO, as well as Cβ nuclei were obtained for the RpfC catalytic domain (RpfCc) using inter-residue connectivities observed in standard triple-resonance experiments. The analysis of Hα and Cα chemical shifts using the Chemical Shift Index Method [35] allowed the initial identification of the secondary structure elements of RpfCc (Fig A in S1 File). Successively, all the side-chain resonances and the NOEs were also assigned. The NOE diagram confirmed the position and length of the secondary structure elements within RpfCc sequence (Fig B in S1 File).

Overall, the data indicated that the catalytic domain presents three helical region spanning residues Trp9-Glu16, Pro37-Gly44, Arg54-Glu68, in agreement with the X-ray structure [11].These findings are a first strong indication that the structure adopted in solution by RpfCc is similar to the structure obtained via X-ray.

**Fig 1:**
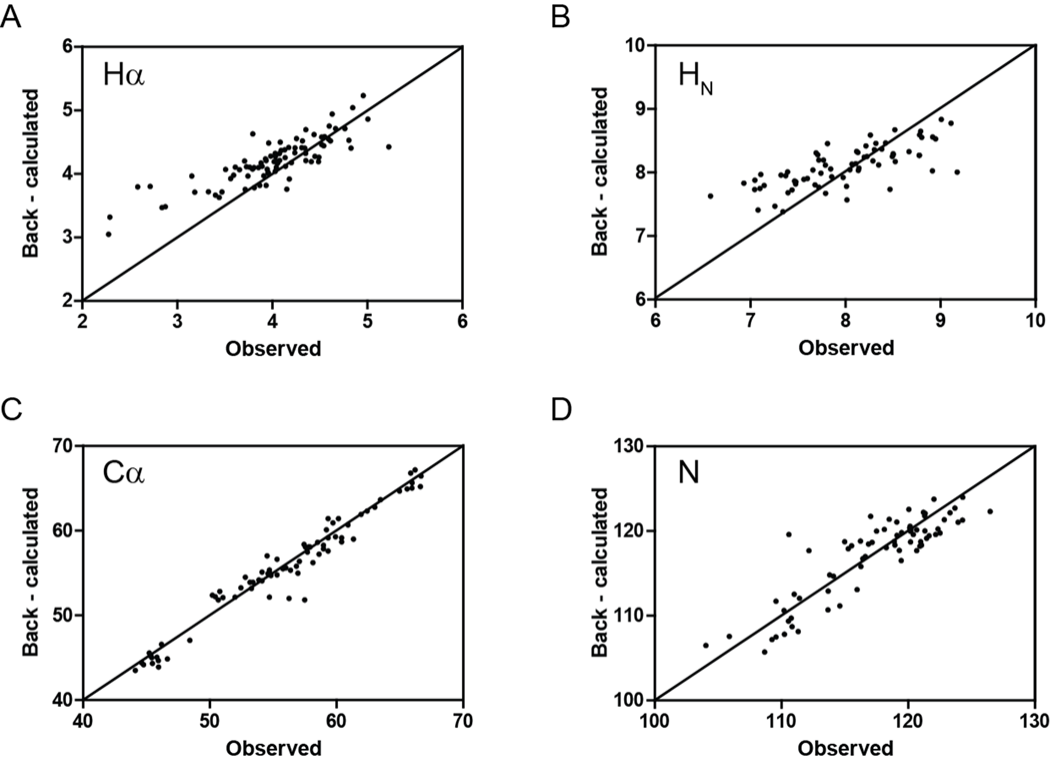
Chemical Shift analysis. Back-calculated versus observed backbone ^1^Hα (A), ^1^H_N_ (B) ^13^Cα (C) and ^15^N (D) Chemical Shifts for RpfCc. The evaluation was performed using the X-ray structure of the catalytic domain of RpfC (PDB code 4OW1).

The crystallographic structure of the catalytic domain of RpfC (PDB code 4OW1) contains eight molecules in the asymmetric unit (named A, B, E, S, T, U, W, X–Fig C in S1 File). On the basis of the RMSD listed in the Table reported in the supporting information (Fig C in S1 File),molecule E has been selected to be the representative structure for chemical shifts evaluation. The backbone chemical shifts (^13^Cα, ^15^N, ^1^H_N_ and ^1^Hα) were then fitted to the RpfCc crystal structure (Fig 1), as reported in the Material and Methods section. All analyzed chemical shifts provided good quality fits as reflected by Q-factor values 0.020 for ^15^N, 0.056 for ^1^H_N_, 0.024 for ^13^Cα and 0.056 for ^1^Hα. Overall, the results confirmed that the conformations adopted by RpfCc in the crystal belong to the conformational ensemble sampled by the protein in solution.

Interestingly, our chemical shift analysis allowed also the identification of various regions endowed with a wide conformational space and therefore a higher flexibility compared to the rest of the protein. These regions include residues between Asn20-Tyr30, Gly44-Gly47 and Trp73-Thr75. These data fostered the following characterization of the structure and the dynamics behaviour of RpfCc in solution.

### Solution structure of RpfC catalytic domain

**Fig 2:**
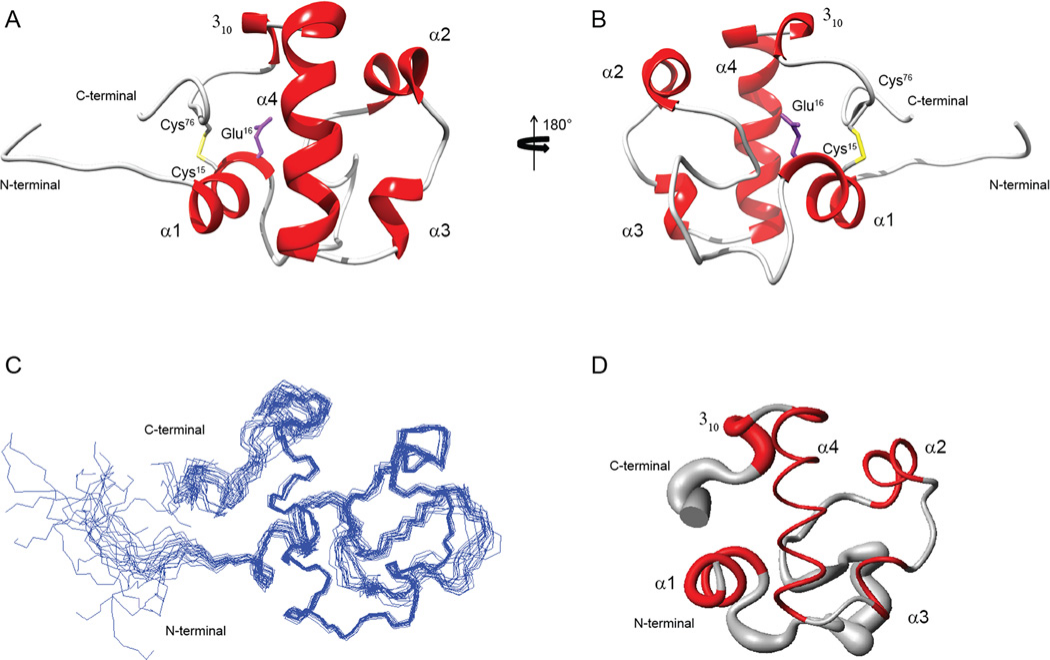
NMR solution structure of RpfCc. (A,B) Ribbon drawing of one representative conformer of the RpfCc NMR structure in two orientation rotated by 180° around z-axes. (C) Overlay of the 20 lowest energy structures of RpfCc. (D) Sausage representation of the RpfCc globular domain (9–78).

The NMR solution structure of RpfCc was calculated, as reported in the Material and Methods section, using 1030inter-proton distance constraints, derived from NOESY experiments, and 120 dihedral angle restraints obtained by TALOS+ software [29] (Table 1). The obtained structure is of high quality and consists of a well structured globular fold of 69 amino acids (with a backbone rmsd of 0.634 Å), ranging from Trp9 to Ala78 and two disordered N and C terminal tails (Fig 2C and 2D). RpfCc presents four α-helices connected by flexible loops to form a helix bundle topology.

The N-terminal flexible tail is constituted by residues Gly1–Pro7 followed by the first α-helix (α1) which is constituted by two turns spanning residues Trp9 to Glu16.This helix contains the catalytic glutamate residue (Glu16) (Fig 2A and 2B), conserved among Rpfs and lysozyme enzymes, and is anchored to the fourth helix by a hydrogen bond between the Nε-H of the Trp9 and the amidic group of Asn62 side chain.

A flexible loop, that ranges from Ser17 to Lys36, connects α1 to the second α-helix (α2). This loop is not well defined as it shows a RMSD_bb_ value of 0.7 Å suggesting that the protein in this region explores a wide conformational space. α2, containing the residues fromPro37 to Phe43, is anchored to the fourth helix (α4) by an hydrogen bond that involves Thr39 side chain-OH and side chain carbonyl group of Gln68 (Fig 2A and 2B).

A five residues loop connects α2 to the short α3 (residues Pro49 to Ala51) while a short two residues loop connects α3 to the fourth helix. α4 is a well defined four turns helix (RMSD_bb_ = 0.16 Å) that ranges from Arg54 to Gln68 and whose axis is nearly orthogonal to α1 and α2 axis. α4 is followed by a short 3_10_ helix in the region Leu70-Ala72.

**Fig 3:**
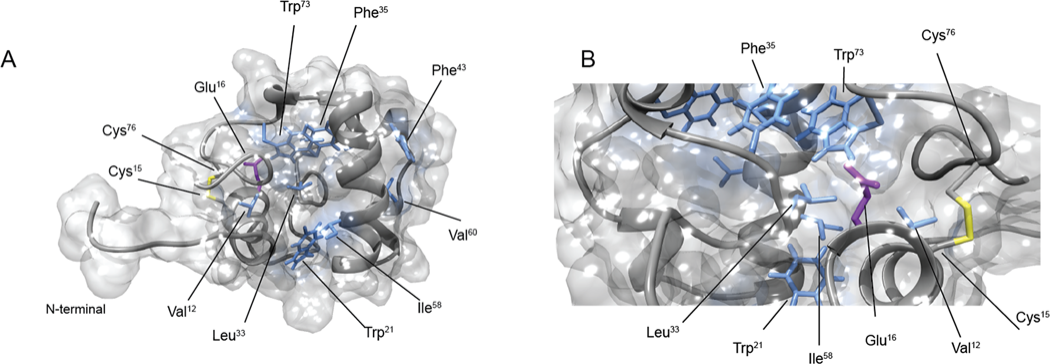
The hydrophobic pocket. (A,B) Close-up views of the hydrophobic pocket close to the catalytic glutamate (Glu16). The catalytic glutamate is depicted in magenta, the disulfide bond (Cys15-Cys76) in yellow and all other residues in light blue.

The C-terminal region does not fold in any predominant secondary structure and is anchored to the N-terminal region of the domain by a disulfide bond (Cys15-Cys76)(Fig 2A and 2B). However, in 4 of the 20 structures of the calculated ensemble, the region Gly77-Ser80 shows an helical propensity. This secondary structure spatial arrangement allows the formation of a compact 9 residues hydrophobic core(Fig 3A).This hydrophobic core is well resolved in the solution structure and is constituted by side chains of residues positioned quite uniformly along the entire domain backbone (Val12, Trp21, Leu33, Phe35, Phe43, Ile58, Val60, Val64, Trp73). In particular, Val12, Trp21, Leu33, Phe35, Ile58, Trp73 constitutes an hydrophobic pocket that is close to catalytic glutamate (Glu16) (Fig 3B). This pocket is well conserved in all Rpfs, suggesting that an hydrophobic environment for the glutamate is important for the catalytic mechanism of these enzymes [36].

### RpfC backbone dynamics probed by Molecular Dynamics simulations

**Fig 4:**
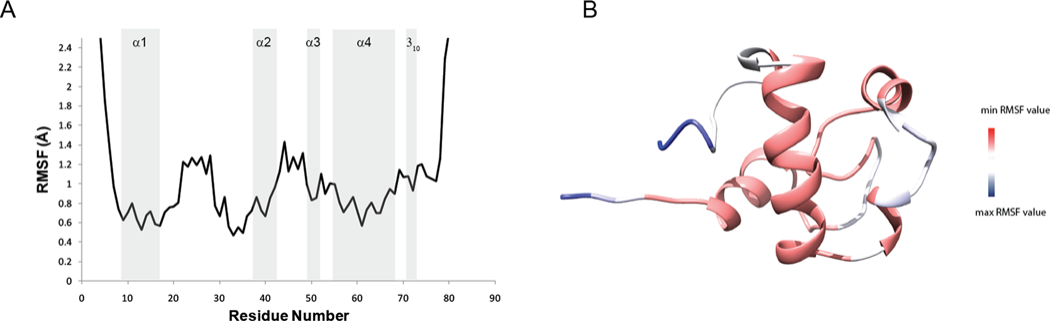
Molecular Dynamics simulation analysis. (A) The Root Mean Square Fluctuations (RMSF) profile of RpfCc of the 60 ns MD simulation versus the residue numbers. (B) RMSF values reported onto the representative conformer of the NMR structure.

In order to describe the dynamic behavior and have a reliable description of the conformational space sampled by RpfCc in solution a set of varied length MD simulations was performed, as described in Materials and Methods. The representative conformer of the calculated NMR ensemble was used as starting structure and the longest MD simulation of 60 ns. In particular, a number of stereochemical parameters (gyration radius, secondary structure, and RMSD) were monitored along the trajectories to evaluate the conformational variations of the RpfCc domain in the simulation timescale. Overall, the trend of these parameters indicates that the simulation converged in ∼10ns. The Root Mean Square Fluctuations (RMSF) profile (Fig 4A and 4B) confirmed that RpfCc adopts a compact module in the region 9–78 with a significant flexibility (RMSF larger than 1.0 Å) in the loop regions. In particular, the three loops l_1_(Asn20-Tyr30), l_2_(Gly44-Gly47) and l_3_(Trp73-Thr75) display rather high RMSF values.

In a recent work, the dynamic behavior of RpfB catalytic domain (RpfBc) was described by using MD simulations [36].The comparison of the MD simulations results obtained for RpfB and RpfC catalytic domains indicates that the two proteins show a quite similar backbone dynamics behavior. In particular, the globular domain of RpfCc (mean RMSF 0.9 Å) appears to be slightly more flexible with respect to the RpfBc (mean RMSF 0.7 Å); the loops l_1_ and l_2_ display the same conformational plasticity in the fast motion time scale; while the loop l_3_inRpfCc is more rigid than in RpfBc. This latter feature may be due to the presence of the short 3_10_ helix (region Leu70-Ala72) in RpfCc that narrowing the sampled conformational space induces a significant reduction of protein motions.

### NMR protein dynamics characterization

**Fig 5:**
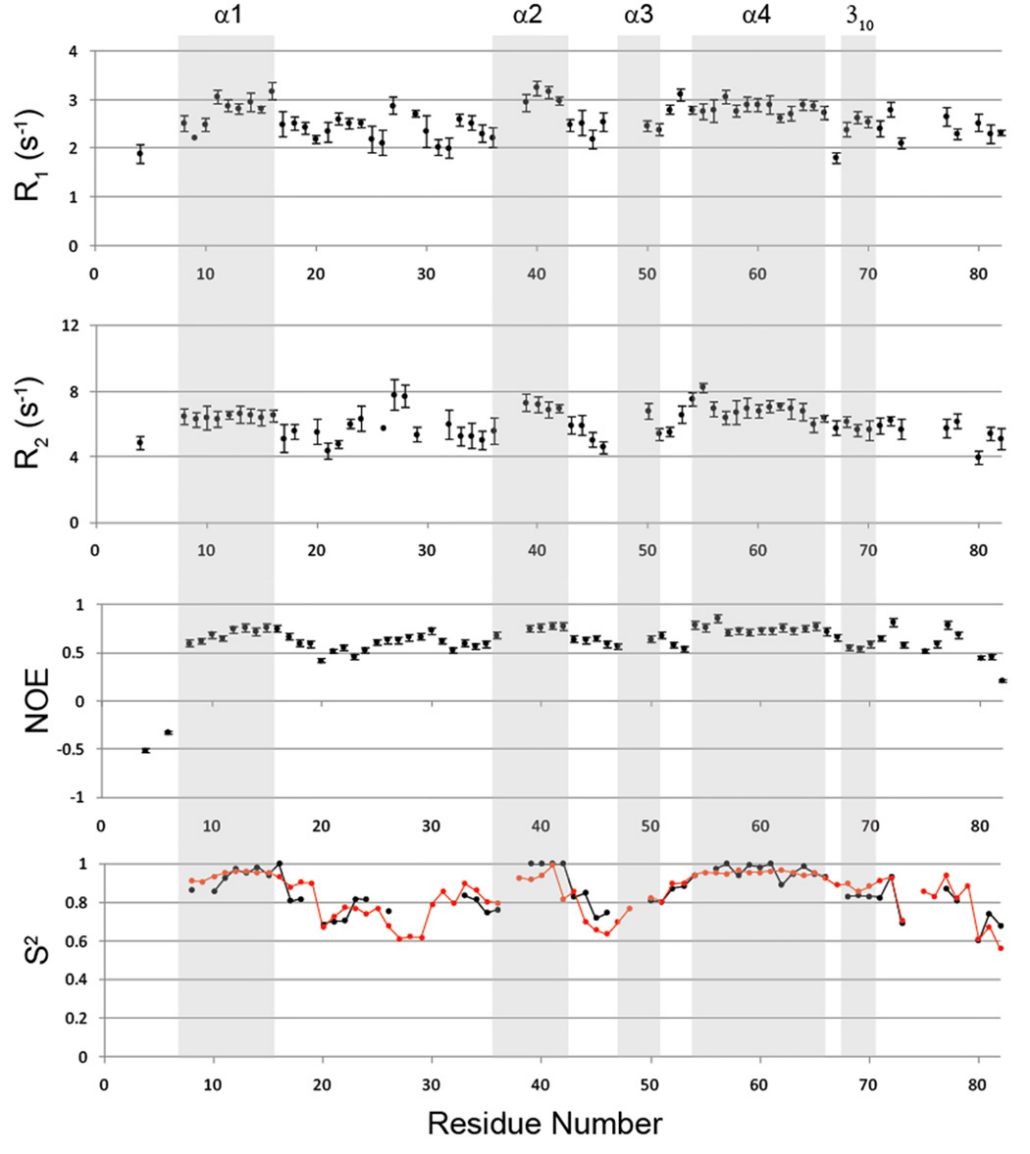
NMR protein dynamics characterization. Relaxation parameters (*R*_1_, *R*_2_, and ^15^N-{^1^H}-NOE) and the order parameter S^2^ (black circle) of RpfCc plotted versus the residue numbers. The order parameter S^2^ derived from the MD data is also reported (red circle).

Since the quality of the MD simulations strongly depends on the applied force field, we performed a cross-validation using experimental NMR relaxation parameters. We characterized three relaxation parameters for each backbone amide for the description the protein motion: the ^15^N R_1_ and R_2_ self-relaxation rates and the steady-state NOE enhancement of the amide ^15^N population due to dipolar cross-relaxation with the attached ^1^H. The two rate constants characterize the decay rate of longitudinal or transverse magnetization and are related to the protein motions on the time scale of pico to nanoseconds (R_1_) or milli to microseconds (R_2_). All relaxation parameters were plotted as function of the amino acid residues and are reported in Fig 5. As expected for a rigid structure, relaxation parameters are generally constant along the whole globular domain (Trp9-Ala78), whereas they are well below the mean values in the N- and C-terminal regions. In particular, the measured relaxation data indicate that the residues located within the three loops (l_1_ Gly18-Gln34, l_2_ Gly44-Gly47 and l_3_ Trp73-Thr75) show a discrete mobility in the picoseconds time scale.

To further characterize the internal mobility of RpfCc, the measured relaxation data were used as input to determine the model-free parameters for each amide group in the protein using the program Dynamics [25, 37]. Five models were used to appropriately fit the dynamical parameters to the experimental relaxation data and the axially symmetric diffusion tensor of the molecule has been chosen as the best fitting the relaxation data. For the axially symmetric model, the rotational diffusion tensor of RpfCc is characterized by D‖/D = 1.3 ± 0.1 and τc = 4.19 ± 0.09 ns (Table 2) which is in excellent agreement with the correlation time derived from the translational diffusion coefficient (τc = 4.17 ± 0.10) (Table 2) obtained by NMR DOSY experiment and from the RpfCc NMR structure analyzed with HYDROPRO software (τc = 4.05 ± 0.27) (Table 2). The obtained S^2^ values (Fig 5) of RpfCc residues are generally high in the globular domain (<S^2^> = 0.87 ± 0.09) indicating that the protein adopts a compact conformation. In particular, the mean values of S^2^ computed for the five helices (including also the short 3_10_ helix Leu70-Ala72) are in the range 0.81–0.97 whereas they slightly drop for the residues located in the three loop l_1_ (<S^2^> = 0.74 ± 0.06), l_2_ (<S^2^> = 0.77 ± 0.07) and l_3_ (<S^2^> = 0.69). Moreover, exchange terms (*R*ex) are required for only four residues (Asn27, Ala50, Trp73 and Leu82) and local correlation times are needed for most of the residues located in the loop regions (Fig D in S1 File).

**Table 2:** Comparison of the experimental correlation times with the values back-calculated for the NMR conformers and the MD ensemble.

Protein	τc(RelaxationNMR data)	τc(DiffusionNMRdata)	τc(HYDRO NMR ensemble)	τc(HYDRO MD ensemble)
RpfCc	4.19 ± 0.09	4.17 ± 0.10	4.05 ± 0.27	4.01 ± 0.52

These finding clearly indicate that the helix regions are relatively rigid whereas the residues located within the loop regions are characterized by a moderate backbone internal flexibility in the picosecond timescale.

In order to check if the NMR analysis is consistent with the Molecular Dynamics data we compared the generalized S^2^ order parameter obtained by the relaxation NMR data (S^2^_NMR_) with the values predicted from MD simulation (S^2^_MD_) (Fig 5). The average S^2^_MD_/S^2^_NMR_ ratio 1.00 ± 0.07 clearly demonstrates that the experimental S^2^ are in perfect agreement with the MD-derived values indicating that the Molecular Dynamics simulation well reproduce the RpfCc backbone dynamics. Additionally, we estimated the correlation time by using the software HYDROPRO [20–22] from the conformers selected along the molecular dynamics trajectory. As reported in the Table 2 the τc predicted from MD data is in good agreement with the correlation time values obtained from the relaxation NMR data as well as from the NMR ensemble.

Overall, the NMR protein dynamics characterization, confirming the Molecular Dynamics simulations, shows that the RpfCc protein presents a rigid globular domain, two flexible tail in the N-terminal and C-terminal region and three loops showing a discrete conformational flexibility.

### Comparing RpfCc structure to other Rpfs

**Fig 6:**
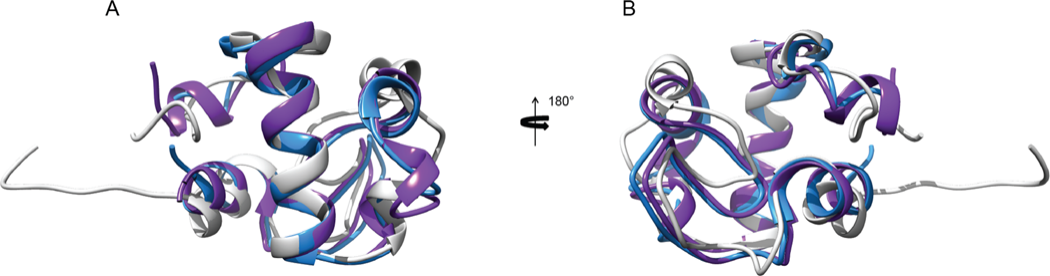
Comparison of RpfCc with other Rpfs. Overlay of the representative conformer of the RpfCc NMR ensemble (gray) with the structure of RpfB (PDB code 3EO5) (magenta) and RpfE (PDB entry 4CGE) (light blue) in two different orientations rotated 180° around z axis.

The RpfC catalytic domain basically adopts the fold already observed in RpfB and RpfE. As expected (Fig E in S1 File) given the 52% sequence identity of the 76 residues, the structural conservation between the RpfCc solution structure and the RpfBc domain is high. The calculated Cα RMSD between the two structures (our structure versus PDB entry 3EO5[8]) is 2.03 Å (Fig 6). When compared to theRpfE structure (PDB entry 4CGE [10], 77 residues with 64% sequence identity) (Fig E in S1 File), the calculated Cα RMSD is 1.93 Å. Most of the backbone geometry is conserved, including the connecting loops between the helices.

Interestingly, a difference is observed within a short sequence insertion, that is present only in the RpfB (Fig 1). A 3_10_-helix (^321^GLRYAPR^327^) is present in RpfBc, while in RpfCc the residues ^45^GVGN^48^, connecting α2 and α3, as well as residues^137^GSGS^140^ in RpfE, show an elongated conformation. This difference results also in a slight change inthe orientation of α-helix 2 within the RPF fold. Moreover, the α-helix in RpfB C-terminal region (Cys355-Arg358) is not well defined in RpfC and RpfE. In particular, as previously discussed, RpfCc shows the presence of this helix only in few structures of the NMR ensemble (4 out of 20).

**Fig 7:**
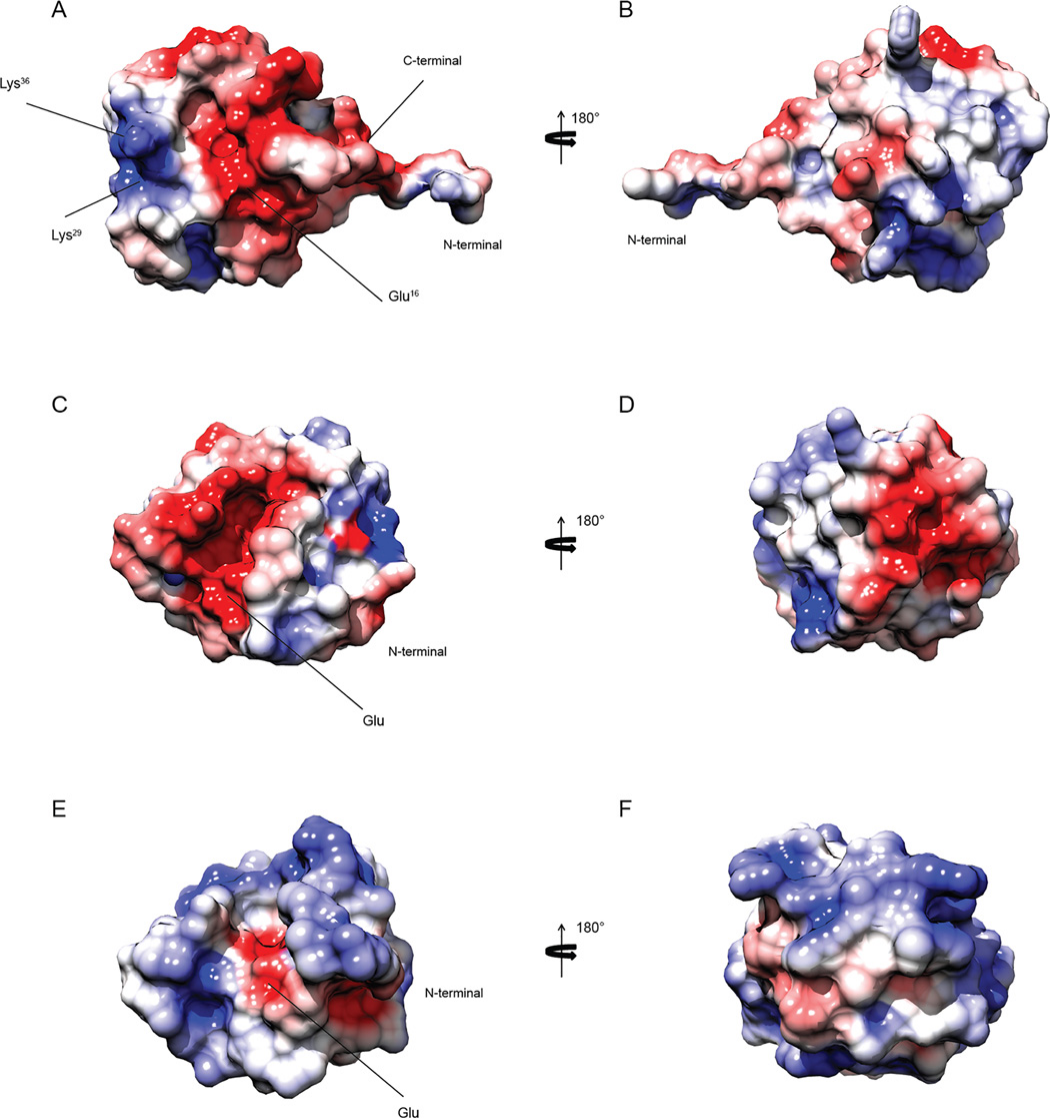
Solvent-accessible surface of RpfCc protein. Electrostatic surface of RpfCc (A,B), RpfBc (C,D) and RpfEc (E,F) in two different orientations rotated 180° around z axis. The positively charged residues are depicted in blue while the negatively charged residues are in red.

The variation in surface charge between RpfB and RpfE has previously been noted [10]. RpfC has two lysines, Lys29 and Lys36, on one side of the catalytic cleft, which are respectively tyrosine and aspartate in RpfB while are leucine and threonine in RpfE. This feature leads to a different charge distribution around the ligand-binding pocket, which may have a role in specificity (Fig 7). In particular, RpfB shows a negatively charged electrostatic potential at the catalytic site, while RpfC and RpfE present a positively charged electrostatic potential with a single negative patch represented by the highly conserved catalytic glutamate residue.

### Structure and stability of RpfCc evaluated by CD spectroscopy

The structural features of RpfCc have been also evaluated via Circular Dichroism (CD) spectroscopy. The spectrum acquired at 25°C is consistent with the NMR structure (Fig F in S1 File) as the pronounced negative peak at 208 nm shows that the protein adopts a well defined fold with a high α-helix content.

**Fig 8:**
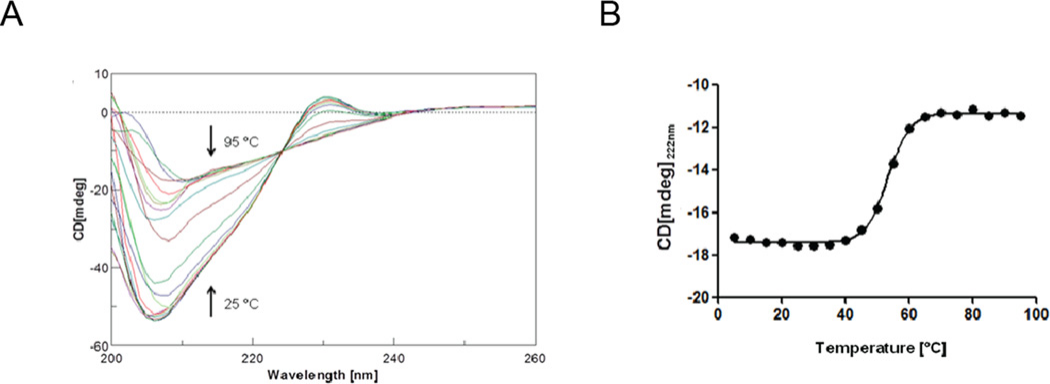
Thermal stability monitored by CD spectroscopy. (A) Thermal unfolding of RpfCc protein followed by circular dichroism. (B) Melting curve monitored by CD at 222 nm. The data were fitted using a two-state model.

The protein has been also characterized in term of stability by performing thermal unfolding experiments encompassing the temperature range between 5°C and 95°C(Fig 8A and 8B). As indicated by the reduction (~7%) of the molar ellipticity at 222nm after cooling back the sample to 25°C the protein unfolding mechanism has been found to be reversible. Therefore, the data werefitted to a classical cooperative two-state model which provided a melting temperature of Tm = 53°C.

Interestingly, the spectra show a positive peak at 230 nm that might either be due to aromatic side chains transitions, in particular of tyrosines and phenylalanines, or due to the n→σ* transition of disulfide bonds[38, 39].

Considering that the NMR and crystal structure of this domain both exhibit the presence of a disulfide bond (Cys15-Cys76), this feature of the CD spectrum appears to be reasonably ascribable to this bond and it has been further analyzed also in the light of the possible role of this interaction in determining the structure and\or the function of this domain.

### Modulation of RpfC function via a redox-sensing switch?

Redox-sensing molecular switches function as unique biological machineries that modulate the functional properties of redox-regulated enzymes, transcriptional factor and sensor proteins. Depending on the redox status, the redox-sensing cysteines can directly modulate the protein function via the formation or hydrolysis of a disulfide bond. Such mechanism, named as "locking and unlocking" may either induce a topology change or indirectly modulate the protein function by influencing distant crucial residues via allosteric-like conformational changes.

**Fig 9:**
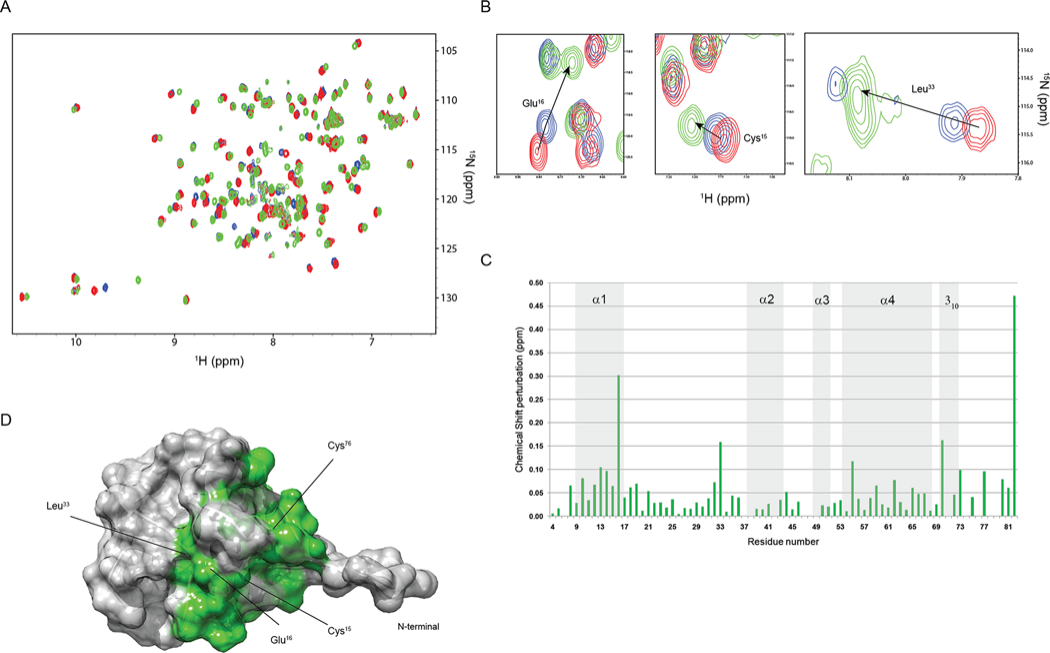
Structural rearrangement of RpfCc monitored by NMR spectroscopy. (A, B) Overlay of the ^1^H-^15^N HSQC spectra acquired at pH 7 (red), pH 5 (blue) and pH 3 (green). (C) Chemical shift changes (ppm) plotted versus the primary sequence. (D) Chemical shift perturbation mapping onto the RpfCc NMR structure.

RpfCc NMR structure shows that the disulfide bond between the two cysteine residues Cys15 and Cys76 is located on the surface of the protein, adjacent to the catalytic site. As such, it is a potential modulator of the protein function. Cys15 is contained within the first α-helix while Cys76 is located in the unstructured C-terminal tail. In order to understand the role played by this disulfide bond on the protein structure we performed an accurate structural investigation using a combination of NMR and CD data. To this aim, we acquired a set of CD spectra of the protein in reducing conditions and at different acidic pHs. The CD spectrum acquired at pH 7 in the presence of TCEP(Fig G in S1 File) shows a similar profile to that obtained in non-reducing conditions, indicating that the secondary and tertiary structure of the protein is preserved. Interestingly, at pH 2 in the presence of TCEP the peak at 230 nm disappears while the protein appears to be still folded. As already mentioned, this peak is typically due either to aromatic side chains transitions or to the presence of the disulfide bridge[38–40]. NMR experiments performed in the same conditions indicate that this change can be ascribed to the hydrolysis of the disulfide bridge between Cys15 and Cys76. Indeed, the ^1^H-^15^N heteronuclear single-quantum coherence (HSQC) spectrum indicates that RpfCc shows a good dispersion of signals in both proton and nitrogen dimensions with the chemical shift of the aromatic residues of the protein only slightly perturbed, witnessing a folded conformation of the protein also at pH 2 in presence of TCEP. Importantly, the backbone chemical shifts perturbation analysis reveals the largest perturbations for residues surrounding the disulfide bond (Gln16, 82). Also Leu33, Glu55 and Leu7 exhibit significant chemical shift changes. These residues that in the primary sequence of the protein are far beyond the cysteines constituting the disulfide bridge, are located in the catalytic pocket (Fig 9).

Overall, these data suggest that the cysteines oxidation state is likely to modulate the shape, in terms of size and volume, of the hydrophobic catalytic cleft, probably modulating the protein function via a protein conformational change mechanism.

## Conclusions

The mechanism through which dormant *M*. *tuberculosis* resuscitates and enters the cell cycle is of tremendous medical interest.

The family of proteins, known as resuscitation-promoting factors (RPFs), has been found to be responsible for bacteria resuscitation and normal proliferation, although Rpfs precise mechanism of action remains unexplained. *M*. *tuberculosis* possesses five RPF paralogues (RpfA–E); they all contain a conserved catalytic domain that shows a variability in composition also found in other species[41]and the presence of N-terminal signal sequences.

Initial functional studies showed that the mycobacterial RPF proteins are redundant, yet phenotypic alterations in the bacterial pathogen appear when deleting three or more *rpf* genes revealing a functional hierarchy of the mycobacterial Rpf proteins [3, 4, 42, 43]. The Rpf catalytic domains show a high degree of structural conservation within the members of the mycobacterial resuscitation-promoting factor family. Possible functional differences among the various Rpf paralogues in the specificity for different peptidoglycan modifications appears then to be based on the composition of the N-terminal portions of the proteins and on small structural changes within RPF catalytic domains such as variation in charge around the active site.

As in protein-ligand interaction processes dynamics is fundamental for the function of the protein, by reporting the structural and dynamic features of *M*. *tubercolosis* RpfCc and its comparison with the other characterized members of this redundant protein family, we aimed at contributing insights in the properties of resuscitation factors.

In this article we show by evaluation of the previously reported x-ray structure using NMR data that the globular domain of RpfCc observed in the crystal is mainly preserved in solution except for the loop regions in which the protein is characterized by a discrete conformational plasticity. To appreciate the functional proprieties of RpfCc, understanding the connection between the three-dimensional structure and dynamics, we performed a backbone dynamics study and a conformational analysis of RpfCc using NMR and MD simulations techniques. Our data confirmed that the globular domain of RpfCc is mainly rigid except for the loop regions, which present a moderate conformational flexibility, and the fully flexible N- and C-terminal tails.

Interestingly, the mobility of the third loop differentiates RpfCc from RpfBc: in RpfCc a short 3_10_ helix (region Leu70-Ala72) induces a significant reduction of protein motions with respect to the same region of RpfBc.

To get insight the functional proprieties of RpfCc we investigated the conformational changes upon pH variation. Our chemical shift perturbation analysis combined with CD data suggests that the formation of the disulfide bridge and thus the cysteines oxidation state is likely to modulate the shape of the hydrophobic catalytic cleft. This might result in a protein function regulation via a “conformational editing” through a disulfide bond formation within a folded domain. It is known that the disulfide bridge is highly conserved within the Rpf family and that replacement of one or both of the cysteine residues impaires[44] Rpfs muralytic activity.

Overall we provide a first glimpse into the relationship between the cysteines oxidation state and the conformational changes associated with it in one of the Rpfs. Comparisons with the other Rpf family members might bring into light differences among the Rpfs that could contribute to the explanation of the functional hierarchy of the mycobacterial Rpf proteins.

Understanding the conformational, structural and functional peculiarities of the different Rpfs is crucial to the design of vaccine and therapeutic strategies.

## Supporting Information

S1 File Chemical Shift Index of the RpfCc protein based on the Cα, Cβ and Hα chemical shifts **(Fig A).** NOE diagram of the RpfCc protein **(Fig B).** The root mean square distributions for backbone heavy atoms of the residues 9–78 with respect to the mean coordinate position between each model reported in the X-ray structure **(Fig C).** The τe and Rex defining the backbone dynamics of RpfCc are plotted as a function of the residue numbers **(Fig D).** (Upper) Multiple alignment ofthe RPF domains (RpfA, RpfB, RpfC, RpfD and RpfE). (Lower) Identity–similarity matrix of Rpf sequences **(Fig E).** CD spectrum of RpfC catalytic domain at pH 7.0 (Fig F). CD spectra of RpfC catalytic domain at pH 7.0 (blue), 5.0 (green) and 2.0 (red) with 2mM TCEP **(Fig G).**
(DOCX)
