# Supplementary material for: NMR Structure and Dynamics of the Resuscitation Promoting Factor RpfC Catalytic Domain
Source: PLoS One. 2015 Nov 17;10(11):e0142807. doi: 10.1371/journal.pone.0142807 (PMC4648573; doi:10.1371/journal.pone.0142807)
Supplement: S1 File — Chemical Shift Index of the RpfCc protein based on the Cα, Cβ and Hα chemical shifts (Fig A). NOE diagram of the RpfCc protein (Fig B). The root mean square distributions for backbone heavy atoms of the residues 9–78 with respect to the mean coordinate position between each model reported in the X-ray structure (Fig C). The τe and Rex defining the backbone dynamics of RpfCc are plotted as a function of the residue numbers (Fig D). (Upper) Multiple alignment ofthe RPF domains (RpfA, RpfB, RpfC, RpfD and RpfE). (Lower) Identity–similarity matrix of Rpf sequences (Fig E). CD spectrum of RpfC catalytic domain at pH 7.0 (Fig F). CD spectra of RpfC catalytic domain at pH 7.0 (blue), 5.0 (green) and 2.0 (red) with 2mM TCEP (Fig G). (DOCX) [file pone.0142807.s001.docx]

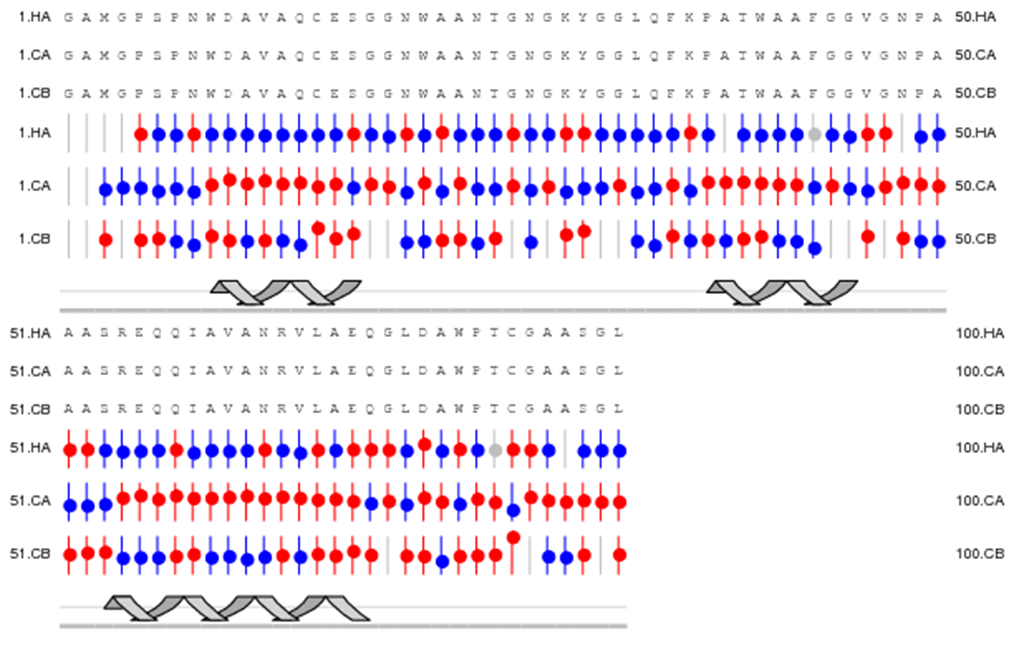


**Figure A.** Chemical Shift Index of the RpfCc protein based on the Cα, Cβ and Hα chemical shifts.


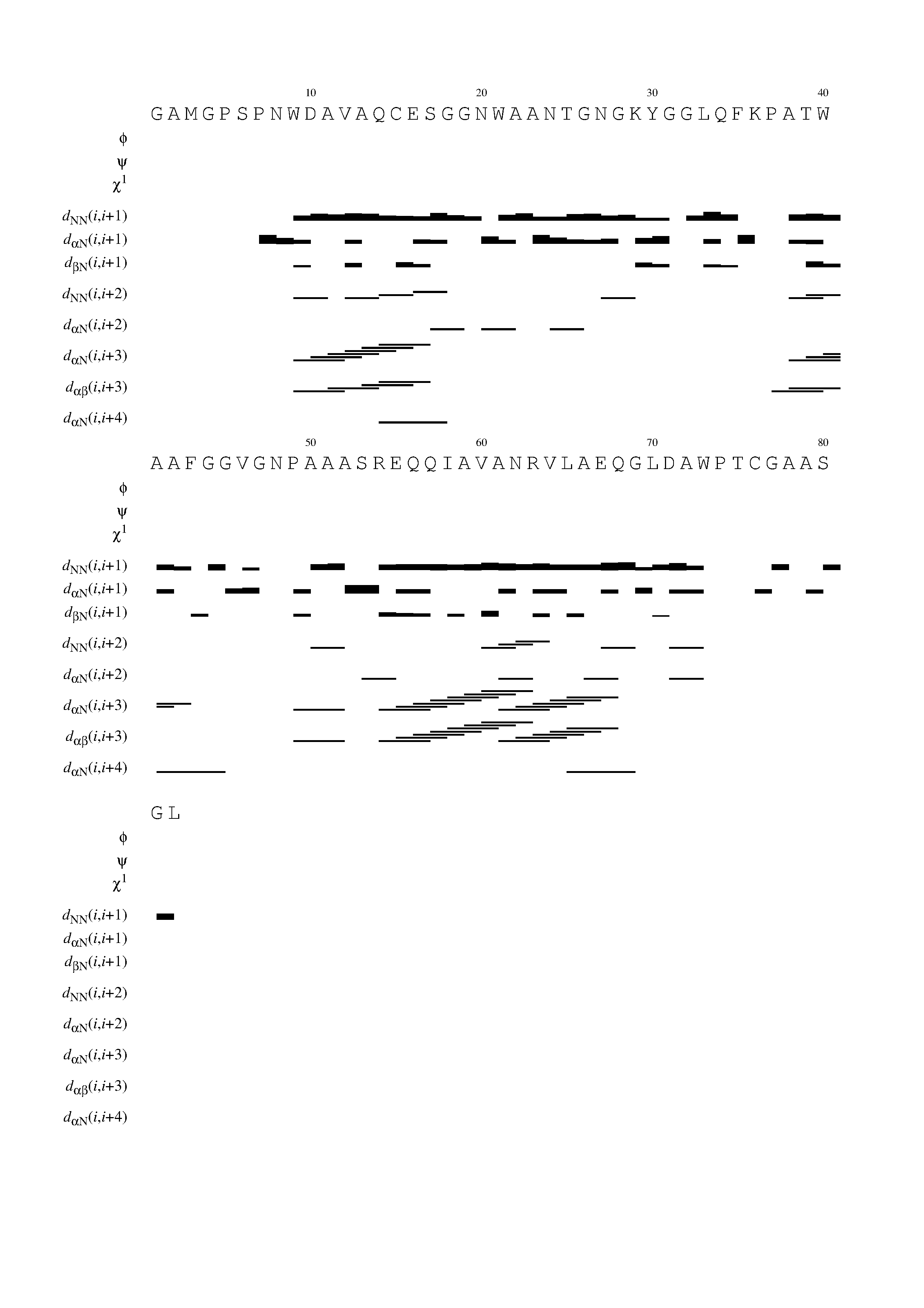


**Figure B.** NOE diagram of the RpfCc protein.

| **MODEL** | **A** | **B** | **E** | **S** | **T** | **U** | **W** | **X** |
| --- | --- | --- | --- | --- | --- | --- | --- | --- |
| **A** |  | 0.078 | 0.091 | 0.139 | 0.155 | 0.088 | 0.106 | 0.113 |
| **B** | 0.078 |  | 0.082 | 0.144 | 0.14 | 0.09 | 0.106 | 0.106 |
| **E** | 0.091 | 0.082 |  | 0.132 | 0.128 | 0.074 | 0.087 | 0.093 |
| **S** | 0.139 | 0.144 | 0.132 |  | 0.093 | 0.146 | 0.124 | 0.098 |
| **T** | 0.155 | 0.14 | 0.128 | 0.093 |  | 0.149 | 0.121 | 0.101 |
| **U** | 0.088 | 0.09 | 0.074 | 0.146 | 0.149 |  | 0.096 | 0.108 |
| **W** | 0.106 | 0.106 | 0.087 | 0.124 | 0.121 | 0.096 |  | 0.078 |
| **X** | 0.113 | 0.106 | 0.093 | 0.098 | 0.101 | 0.108 | 0.078 |  |

**Figure C.** The root mean square distributions for backbone heavy atoms of the residues 9–78 with respect to the mean coordinate position between each model (A-X) reported in the X-ray structure.

**
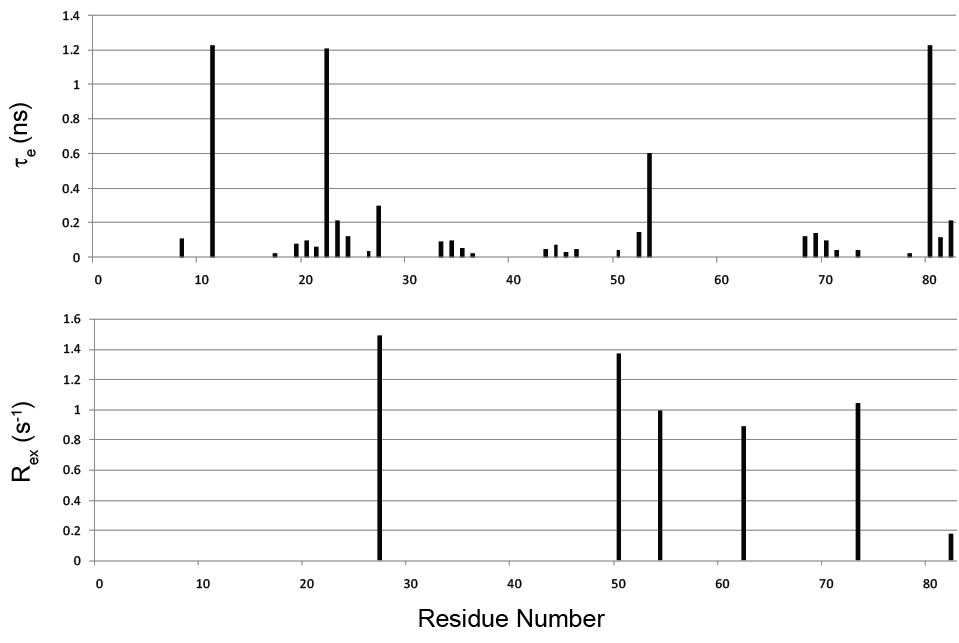
**

**Figure D.** The τe and R_ex_ defining the backbone dynamics of RpfCc are plotted as a function of the residue numbers.


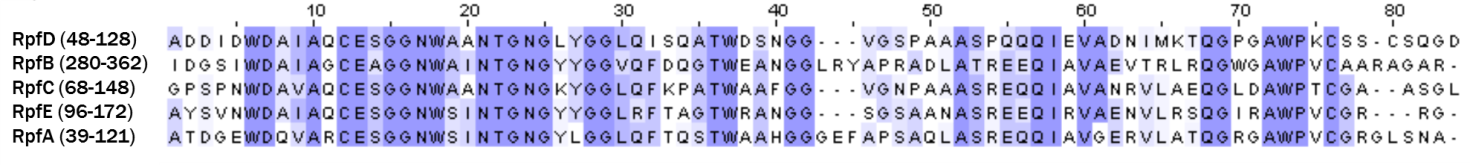


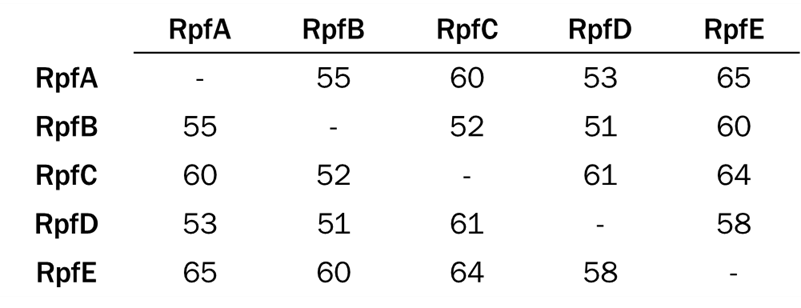


**Figure E**. (Upper) Multiple alignment ofthe RPF domains (RpfA, RpfB, RpfC, RpfD and RpfE). (Lower) Identity–similarity matrix of Rpf sequences.

**
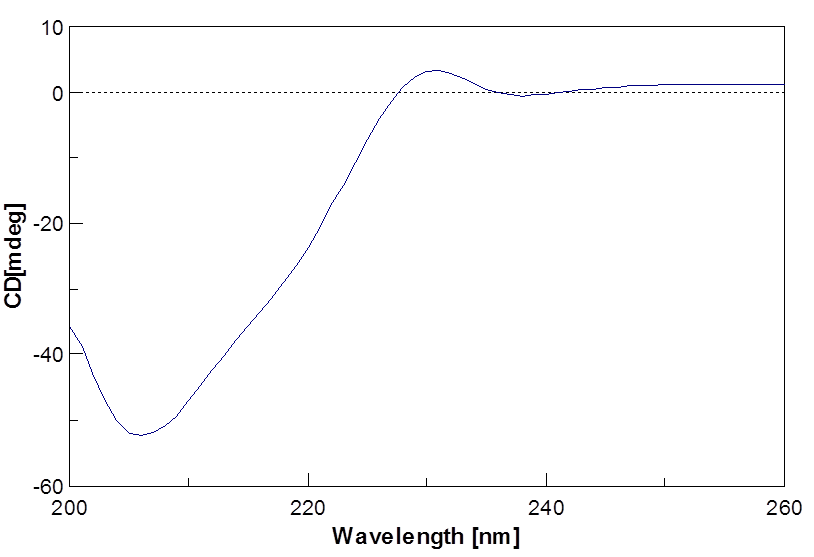
**

**Figure F.** CD spectrum of RpfC catalytic domain at pH 7.0.

**
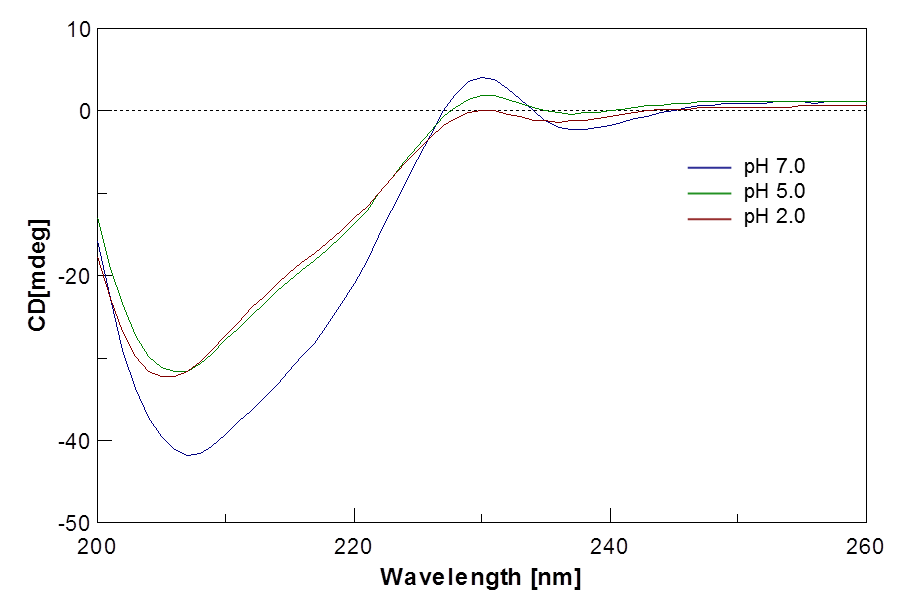
**

**Figure G.** CD spectra of RpfC catalytic domain at pH 7.0 (blue), 5.0 (green) and 2.0 (red) with 2mM TCEP.
